# Female genital mutilation/cutting and birthing: Enhanced education and training is critical for health care providers

**DOI:** 10.7189/jogh.12.03059

**Published:** 2022-09-03

**Authors:** Jacob M Lurie, Tara Pilato, Gunisha Kaur

**Affiliations:** 1 NewYork-Presbyterian Hospital, New York City, New York, USA; 2 Temple University Hospital, Philadelphia, Pennsylvania, USA; 3 Department of Anesthesiology, Weill Cornell Medicine, New York City, New York, USA

While the World Health Organization calls for a universal ban on female genital mutilation/cutting (FGM/C), 200 million women and girls around the world are affected by the ongoing practice [1]. Physicians in high-income countries will encounter patients with FGM/C more frequently. For example, in 2021, over half of the top ten countries of origin for refugees admitted to the United States practised FGM/C, with some nations having a prevalence of FGM/C of nearly 90% [2]. Refugee admissions are also set to increase from a maximum of 62 500 in fiscal year 2021 to 125 000 for fiscal year 2022 [3]. This is added to the 44 million immigrants residing in the United States and the over three million refugees admitted since 1975. However, FGM/C remains an enigma for providers who often do not recognize its signs and symptoms, do not understand how to query patients for its known obstetric and gynecologic complications, and do not know how to meet the unique health needs of these women and girls [4].

Most of those who undergo FGM/C are children. It is estimated that half of the incidences of FGM/C occur in children under five years of age, and most other cases occur in children five to fifteen years of age [4]. Experiencing trauma, especially during childhood, is known to increase the risk of numerous long-term health problems such as cardiac, pulmonary, and liver disease, psychiatric illnesses such as depression and anxiety, and rates of illicit drug and alcohol abuse [5-7]. With this understanding, we propose a trauma-informed approach to treating patients who have undergone FGM/C.

## OUR UNDERSTANDING OF FEMALE GENITAL MUTILATION/CUTTING

In over a decade of work, our group at the Weill Cornell Center for Human Rights has seen several dozen patients with FGM/C from around the world. We learned that health care providers often lack an understanding of FGM/C and its long-term health complications. To help fill this gap, our group published one of the most comprehensive systematic reviews and meta-analyses on FGM/C and its painful obstetric and gynecological outcomes [8]. While we found significant associations between the practice and numerous detrimental obstetric and gynecological outcomes, it was the health care providers’ unfamiliarity with FGM/C that amplified the negative effects in patients. A comprehensive survey of several hundred Somali refugees giving birth in Canada after FGM/C, for example, found that the vast majority (nearly nine in 10) reported offensive comments from their caregivers because of their cutting. Two out of five women stated that they would not return to the same hospital for future deliveries; 10% of women reported that they would prefer not to attend any hospital for future births, a particularly poor outcome given the potential birthing complications in this population [9].

**Figure:**
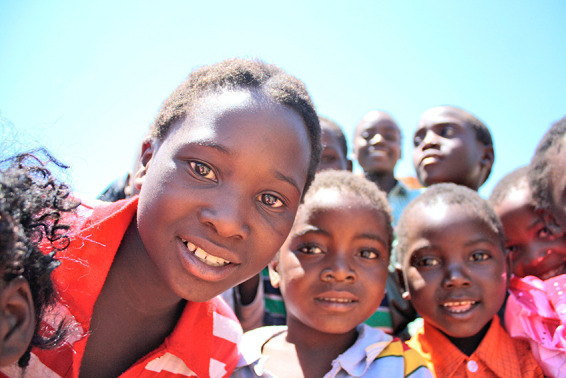
Photo: Free to use under Pexels license. Available at: https://www.pexels.com/cs-cz/foto/muz-lide-zena-afrika-2883380/.

## HOW PHYSICIANS CAN PROCEED

We propose specific actions that should be taken by health care providers when encountering a patient who has undergone FGM/C, rooted in the principles of trauma-informed care. Trauma-informed care recognizes the widespread impact of trauma on all aspects of a patient’s life and promotes a path for recovery [10]. Such an approach should be holistic and patient-centered, acting as a framework to serve patients with nuanced experiences. The Substance Abuse and Mental Health Services Administration (SAMHSA) proposes standards for a trauma-informed approach to patient care. We apply such principles to the care of FGM/C patients in **Table 1**.

**Table 1:** Principles in providing health care to women having undergone female genital mutilation/cutting (FGM/C)

Principle	Context	Methods to improve care
Approach encounter with appropriate knowledge base, and strive to learn more	Healthcare providers should equip themselves with the appropriate cultural competence and medically relevant knowledge concerning FGM/C and its consequences.	Familiarize oneself with FGM/C and its common sequelae to guide treatment plans and aid in trust-building. A working understanding of the practice is crucial for forging physician-patient relationships.
Meet patients where they are	FGM/C is often a traumatizing experience for women, and it can lead to repeat, life-long traumas. However, FGM/C is sometimes highly regarded by those affected by the practice. Physicians should not make assumptions about a patient’s experience or feelings about FGM/C.	Healthcare providers should take a detailed history and inquire as to the patient’s understanding of the practices’ health consequences and whether the patient herself is experiencing any distressing symptoms. Allow patients to tell their own stories and provide supportive, active listening.
Connect patients with resources and ensure collaboration between providers	Often, women who have undergone FGM/C are unaware of resources available to them or how to access these materials.	Healthcare providers should be familiar with referral methods to national or local support groups and resources available through the American College of Obstetricians and Gynecologists, World Health Organization, and Office of Women’s Health at the US Department of Health and Human Services.
Advocate for patients and familiarize oneself with current federal and state-level legislation	It is illegal in the United States to perform FGM/C, and it is illegal for any girl under the age of 18 to be sent outside of the United States for FGM/C to be performed. The United States considers FGM/C to be a form of gender-based violence and child abuse.	Recognize that a woman who has undergone FGM/C is not at fault. Physicians should educate patients on the dangerous consequences of FGM/C and help stop its perpetuation while providing support and treatment to women who have undergone this procedure.

First, physicians must strive to prevent re-traumatization. FGM/C may constitute only a fraction of the distress that a refugee woman experiences: A refugee child who has suffered persecution in her home country, for example, may have also suffered traumas en route to her new host nation, and even within her new country (e.g., family separation or immigration detention). Such traumas can compound and potentiate the psychological burden of children who have already experienced FGM/C.

Rehabilitation should begin with the patient’s first office visit. This initial encounter can impact patient retention and the course of an individual’s care. The office should be welcoming, with minimal unneeded noise. During the medical encounter, the provider should begin by explaining how the encounter will progress. While speaking with the patient, proper interpretation is essential so that nuances are not lost in conversation. Terminology is also critical; although commonly used among physicians, the word “mutilation,” for example, should be avoided due to its negative connotation. During the medical interview, patients may have difficulty describing their symptoms, or have vague complaints such as diffuse abdominal pain – these nebulous details may frustrate physicians in search of an etiology. It is important that the first visit be marked by support, positivity, and trust-building, and this should supersede any initial medical examination or investigation. The extent of one’s FGM/C (ie, Type I, Type II, Type III, or Type IV) does not necessarily correlate with the extent of trauma, and patients who underwent FGM/C should be approached with active empathy.

When performing the physical exam, it is important to grant patients autonomy over their bodies. Ask permission before examining a patient; if a patient requires an invasive procedure, such as a Pap smear, allow for the testing to be postponed until another visit, after the patient is more comfortable with the provider. Patients should be encouraged to follow up regularly, and ideally always with the same physician for continuity. If a patient misses one or more visits, the physician should recognize that this may stem from fear or trauma and not become discouraged. Practice patience and partner with the patient. If specialist follow-up is required, it is useful to recommend providers with experience in trauma-informed care, if such practitioners are available.

## CONCLUSIONS

Healthcare providers are uniquely positioned to address trauma for vulnerable patients, such as those who have undergone FGM/C. The first step is to educate ourselves on what is necessary to meet the health needs of this population. Rehabilitation for survivors of FGM/C should include a long-term partnership between the physician and patient and provide avenues for gradual recovery. A trauma-informed approach can reinforce the sanctity of the doctor-patient relationship, increasing the potential for health care engagement and physical and psychological rehabilitation of women with FGM/C.
